# Whole Genome Sequence of *Bacillus velezensis* Strain GUMT319: A Potential Biocontrol Agent Against Tobacco Black Shank Disease

**DOI:** 10.3389/fmicb.2021.658113

**Published:** 2021-07-06

**Authors:** Haixia Ding, Weidi Mo, Shui Yu, Huanhuan Cheng, Lijuan Peng, Zuoyi Liu

**Affiliations:** ^1^Department of Plant Pathology, College of Agriculture, Guizhou University, Guiyang, China; ^2^Guizhou Academy of Agricultural Sciences, Guiyang, China; ^3^College of Tobacco Science, Guizhou University, Guiyang, China; ^4^Guizhou Key Laboratory of Agricultural Biotechnology, Guizhou Academy of Agricultural Sciences, Guiyang, China

**Keywords:** tobacco black shank, *Bacillus velezensis*, biocontrol, genome sequencing, antifungal

## Abstract

*Phytophthora nicotianae* causes black shank, a serious soil-borne disease, in tobacco. In this study, the *Bacillus* strain GUMT319 was isolated from the rhizosphere of healthy tobacco plants grown in a field in Guizhou with a high incidence of tobacco black shank. Genome sequencing revealed that GUMT319 contained a single circular chromosome 3,940,023 bp in length, with 4,053 predicted genes and an average GC content of 46.6%. Based on phylogenomic analyses, GUMT319 was designated as *Bacillus velezensis*. The genome of GUMT319 contained more than 60 genes and 13 gene clusters that have previously been found to be active in antifungal mechanisms, biofilm formation, and chemotaxis motility. Additionally, confocal laser scanning microscopy and scanning electron microscopy showed that GUMT319 formed a spatially organized biofilm *in vivo*. In addition, lauric acid negatively regulated biofilm formation. This is the first study to report that nicotine in tobacco root exudates was a chemoattractant for biocontrol *Bacillus* strains. In this study, we identified new interactions between beneficial microorganisms and tobacco roots in the rhizosphere. Moreover, dual culture tests *in vitro* showed that GUMT319 inhibited the growth of *P. nicotianae* and also displayed inhibitory effects against eight other plant pathogens, namely, *Colletotrichum scovillei*, *Colletotrichum capsici*, *Fusarium carminascens*, *Sclerotinia sclerotiorum*, *Alternaria alternata*, *Phomopsis* sp., *Phyllosticta sorghina*, and *Exserohilum turcicum*. Furthermore, GUMT319 exhibited > 70% control efficiency against tobacco black shank in field experiments conducted in 2018–2020. Thus, GUMT319 was more effective in controlling the incidence of tobacco black shank than other treatments including fungicide application. Overall, these results suggested that GUMT319 (*B. velezensis*) could be used as a potential biocontrol agent against tobacco black shank.

## Introduction

Tobacco black shank, caused by the fungal pathogen *Phytophthora nicotianae*, is a serious disease of flue-cured tobacco, which is an economically important crop in Guizhou, China. The severity of tobacco black shank continues to increase each year, causing significant economic losses. In the field, tobacco black shank affects the stem base (Han et al., 2016; Guo et al., 2020) and the chemical fungicide metalaxyl is generally used for disease control. However, this practice is not conducive to the development of sustainable tobacco agriculture, as it creates an excessive dependence on chemical agents, leaves fungicide residues, and causes environmental pollution (Han et al., 2016; Guo et al., 2020). As an alternative, biological control agents (BCAs) offer safe alternative methods for controlling tobacco black shank (Zhang et al., 2017a; Srikhong et al., 2018).

Biocontrol has proved to be a promising strategy for the management of many plant diseases (Chowdhury et al., 2015; Han et al., 2016; Zhang et al., 2017c; Fan et al., 2018, Mao et al., 2020). Numerous BCAs have been studied, but only a limited number of strains, such as those of *Bacillus* species, have been commercially developed (Chowdhury et al., 2015; Fan et al., 2018; Ye et al., 2020). *Bacillus* spp. are ubiquitous bacteria widely distributed in natural environments, especially in the rhizosphere and plant roots. They are also excellent candidates for BCAs since they produce heat- and desiccation-resistant endospores that are easily stored and transported as stable products (Weng et al., 2013; Fan et al., 2018). However, the use of BCAs remains a challenge since their effect in the field is frequently inconsistent (Weng et al., 2013).

*Bacillus* spp. employ a number of mechanisms in their effective biocontrol of pathogens, such as antagonism, systemic resistance induction, and plant growth promotion (Chowdhury et al., 2015). In previous studies, *Bacillus* strains exhibited biological activity against various plant pathogens by producing different types of antimicrobial compounds, such as cell wall-degrading enzymes and non-ribosomally synthesized antibiotics, as well as secondary metabolites that trigger induced systemic resistance (ISR), thus protecting the plants against pathogen attack (Chowdhury et al., 2015; Fan et al., 2018; Yan et al., 2020).

Successful root colonization of BCAs is the key to effective biocontrol and is considered to be the main factor responsible for consistent performance in the field (Weng et al., 2013; Xu et al., 2013; Liu et al., 2014). Root colonization is divided into two steps: chemotaxis toward the root and subsequent biofilm formation on the root surface (Zhang et al., 2017b; Feng et al., 2018, 2019). *Bacillus* spp. colonize the roots of many plant species and form a biofilm, which contributes to their biocontrol efficacy (Gao et al., 2015a). Chemotaxis-mediated response to root exudates enhances the colonization and beneficial effects of plant growth-promoting *Bacillus* strains (Feng et al., 2018).

*Bacillus velezensis* is an important member of the plant growth-promoting rhizobacteria (PGPR), which are known to enhance plant growth and control soil-borne diseases (Chowdhury et al., 2015; Fan et al., 2018). *B. velezensis* FZB42 was the first strain to be sequenced (Chen et al., 2007). It was initially identified as a *Bacillus amyloliquefaciens* strain, but was later recognized as a *B. velezensis* strain and as a model of gram-positive plant growth-promoting and biocontrol rhizobacteria (Fan et al., 2018). Approximately 130 whole genome sequences of *B. velezensis* have been deposited in GenBank to date. *B. velezensis* strains have their own specific genomic characteristics, because these strains reside in different host plants and environments (Wang et al., 2019). For instance, *B. velezensis* SQR9 isolated from the cucumber rhizosphere has been used as a BCA against fungal pathogens, mainly because of its ability to trigger ISR (Borriss et al., 2018; Wu et al., 2018), while *B. velezensis* HAB-2 isolated from cotton has been used as a biological pesticide against bacterial pathogens (Xu et al., 2020).

In this study, we isolated *B. velezensis* GUMT319 from the rhizosphere of healthy tobacco plants growing in high-incidence tobacco black shank fields in Guizhou, China. Previous research has shown that GUMT319 produces enzymes with biocontrol activity, such as proteases, cellulases, siderophores, and phosphatases, and inhibits the mycelial growth of *P. nicotianae in vitro* (Luo et al., 2019). Here, we aimed to investigate the biocontrol effects of GUMT319 in the field and to gain insights into the underlying mechanisms. Genome sequencing of GUMT319 revealed the presence of genes involved in plant growth promotion, biofilm formation, chemotaxis, and antifungal activity. In addition, confocal laser scanning microscopy and scanning electron microscopy analyses of the GUMT319 strain labeled with green fluorescent protein (GFP) revealed its patterns of tobacco root colonization. Overall, this study highlights the excellent potential of GUMT319 as a BCA against the tobacco black shank disease.

## Materials and Methods

### Strains and Culture Conditions

*Bacillus* strain GUMT319 was grown at 37°C in Luria–Bertani medium (LB). The GFP-labeled GUMT319 (GUMT319-gfp) was maintained in LB supplemented with 10 mg/mL tetracycline. The fungal pathogens used in this study included *P. nicotianae*, *Colletotrichum scovillei*, *Colletotrichum capsici*, *Fusarium carminascens*, *Sclerotinia sclerotiorum*, *Alternaria alternata*, *Phomopsis* spp., *Phyllosticta sorghina*, and *Exserohilum turcicum*. These were maintained on potato dextrose agar (PDA) medium at the Department of Plant Pathology, Guizhou University. To store these pathogens, hyphae were sampled from the growing zone of mycelia in agar disks (1 cm diameter), and they were mixed with 15% glycerol and stored in a 4°C freezer.

### Sequencing and Phylogenetic Analysis

A single bacterial colony was inoculated in 5 mL of LB broth and grown for 12 h at 37°C with agitation at 200 rpm. Then, 2 mL of the bacterial culture was centrifuged at 10,000 rpm for 1 min, and bacterial genomic DNA was isolated using an Ezup Column Bacteria Genomic DNA Purification Kit [Sangon Biotech (Shanghai) Co., Ltd., China], in accordance with the manufacturer’s protocol. Subsequently, *16S rRNA* and *gyrA* genes were amplified by PCR using sequence-specific primers: 27F (5′-AGAGTTTGATCCTGGCTCAG-3′) and 1492R (5′-GGTTACCTTGTTACGACTT-3′) for amplifying approximately 1,400 bp of the *16S rRNA* gene and *gyrA*-F (5′-CAGTCAGGAAATGCGTACGTCCTT-3′) and *gyrA*-R (5′-CAAGGTAATGCTCCAGGCATTGCT-3′) for amplifying approximately 1,000 bp of the *gyrA* gene (Chen et al., 2016). Each 25-μL PCR mixture contained 1 unit of Pfu DNA Polymerase (Sangon Biotech), 1 × PCR buffer, 1 mM MgCl_2_, 100 μM dNTPs, 1 μM of each primer, 50 ng of genomic DNA template, and ultrapure water. PCR was performed using the following conditions: initial denaturation at 95°C for 10 min, followed by 30 cycles of denaturation at 94°C for 30 s, annealing at 56°C for 30 s, and extension at 72°C for 90 s, with a final extension at 72°C for 10 min. The PCR products were sequenced at Sangon Biotech. Sequences of the *16S rRNA* and *gyrA* gene fragments were searched in the National Center for Biotechnology Information (NCBI) nucleotide database using Blastn to determine the closest taxonomic relatives. Subsequently, phylogenetic analysis of *16S rRNA* and *gyrA* gene sequences was performed in MEGA 6.0 using the maximum likelihood method to estimate the evolutionary position of GUMT319 relative to other *Bacillus* strains (Supplementary Table 1).

### Genome Sequencing and Annotation

The genomic DNA of GUMT319 was sequenced at Beijing Novogene Bioinformatics Technology Co., Ltd. using an Illumina PE150 system and PacBio RSII high-throughput sequencing technology. Low-quality reads were filtered using SMRT Link v5.0.1, and the filtered reads were assembled into one contig without gaps. The complete genome sequence of GUMT319 was annotated using the Prokaryotic Genomes Annotation Pipeline (PGAP) at NCBI, and gene functions were predicted using five databases, namely, Gene Ontology (GO), Kyoto Encyclopedia of Genes and Genomes (KEGG), Clusters of Orthologous Groups (COG), Non-Redundant (NR) protein sequences, and Swiss-Prot, based on whole genome Blast search (*E*-value < 1e-5; minimal alignment length > 40%) against each database.

### Construction of GFP-Labeled GUMT319

The *GFP* plasmid pGFP78, an *Escherichia coli*–*Bacillus subtilis* shuttle vector containing the 78 promoter-controlled *GFP* gene (Zhang et al., 2017c), was electroporated into electrocompetent cells of strain GUMT319, as described previously (Zhang et al., 2012). Briefly, the cells were grown in liquid LB medium at 37°C and were harvested during the exponential growth phase by centrifugation at 10,000 rpm for 10 min at 4°C. The harvested cells were washed five times with an equal volume of cold electroporation buffer containing 0.5 M sorbitol, 0.5 M mannitol, and 10% glycerol (pH 7.0). Subsequently, 100 μL of competent cells were electroporated on ice with 1 mg of pGFP78 DNA using a 1.8-kV electric shock. After electroporation, the cell suspension was diluted with 900 μL of LB medium and incubated at 37°C on an agitator at 120 rpm for 3 h to allow the expression of antibiotic resistance markers. The cell suspension was then spread on LB agar medium supplemented with tetracycline (10 mg/mL). The GFP-labeled cells were selected for tetracycline for three generations, and this was confirmed by fluorescence microscopy (Gao et al., 2015b).

### Plant Material and Growth Conditions

Seeds of tobacco (*Nicotiana tabacum*) cultivar Yunyan 87, native to China, were surface-sterilized by soaking in 2% sodium hypochlorite for 15 min and then washed thoroughly with distilled water. The sterilized seeds were sown in axenic tissue culture bottles containing vermiculite, and seedlings grew for 15–20 days in a growth chamber at 25°C day/20°C night temperature and a 16-h light/8-h dark photoperiod. Then, seedlings were aseptically transplanted into 250-mL flasks (one seedling per flask) containing 100 mL sterile liquid 25% sucrose-free Murashige and Skoog (MS) medium, which was renewed every other day during the growth period. The hydroponic system was placed on an agitator and gently shaken at 50 rpm for 2 h each day. Before inoculating the MS medium with *B. velezensis* GUMT319-gfp, 100-μL aliquots of the MS medium were sampled from each flask and spread onto solid LB medium to verify the absence of contamination (Han et al., 2016).

### Root Colonization Assay

To perform the root colonization assay, GUMT319-gfp was grown overnight in liquid LB medium and was subsequently resuspended in sterile double-distilled water. Roots of tobacco plants were inoculated with 100 mL of GUMT319-gfp (OD_600_ = 0.5) *via* drench application for 2 h; 10 replicates were performed. The plants were then transferred to fresh MS medium and cultivated for an additional 2 days without agitation. Roots were rinsed with sterile double-distilled water. To visualize colonization by GUMT319-gfp and GUMT319, at least 20 root tips were observed under a confocal laser scanning microscope (CLSM) and scanning electron microscope (SEM), as described previously (Weng et al., 2013).

### Collection and Analysis of Root Exudates

Seeds of tobacco cultivar Yunyan 87 and pepper cultivar Dangwu were germinated, and the seedlings were grown for approximately 15–20 days in MS medium, as described above. Before starting the collection of root exudates, plant roots were washed with sterile double-distilled water to avoid contamination from the nutrient solution. The plants were then transferred to a flask, with the roots submerged in sterile water, and the flasks were placed in a growth chamber for 24 h at 25°C and a 16-h light/8-h dark photoperiod, with gentle agitation (50 rpm). The root exudates collected were filtered through a 0.45-μm membrane and lyophilized. The freeze-dried powder of tobacco root exudates was dissolved in ethanol and concentrated 50-fold. To ensure that the root exudates were free from contamination, 100 μL of the filtered root exudate was plated on LB agar medium, and the plates were incubated at 30°C for 24 h.

GC-TOF-MS (Nanjing Zoonbio Biotechnology Co., Ltd., Nanjing, China) was performed to qualitatively analyze the freeze-dried root exudates using an Agilent 7890 gas chromatograph system coupled with a Pegasus HT time-of-flight mass spectrometer. Chroma TOF 4.3X software (LECO Corporation, St. Joseph, MI, United States) and the LECO-Fiehn Rtx5 database were used to extract raw peaks, data baseline filtering, and calibration of the baseline, peak alignment, deconvolution analysis, peak identification, and integration of the peak area. Both the mass spectrum match and retention index match were considered during metabolite identification.

### Motility Assay

*B. velezensis* GUMT319 was grown in LB broth until reaching an OD_600_ of 0.8. Subsequently, 10 mL of the culture was centrifuged. The cell pellet was washed twice with distilled water and resuspended in 100 μL. Then, 15 mL of LB (0.7% agar) medium was poured into a Petri dish (90 mm diameter), and a 5-μL drop of concentrated bacterial culture was dispensed at the center of the dish. The Petri dish was incubated at 37°C, and the appearance of the bacterial zone was observed after 12 h (de Weert et al., 2002).

### Chemotaxis Assay

*B. velezensis* GUMT319 was grown and harvested as described above. Then, 15 mL of LB (0.7% agar) medium with the 50-fold concentrated root exudates (or 200 μM different composition of root exudates) was poured into a 90-mm-diameter Petri dish. A 5-μL drop of concentrated bacterial culture was added to the center of the dish and incubated at 37°C for 12 h to check for the appearance of the bacterial zone (de Weert et al., 2002; Weng et al., 2013).

### Biofilm Formation Assay

The biofilm formation efficiency of *B. velezensis* GUMT319 was quantified using the microtiter plate test. Briefly, GUMT319 was grown in 5 mL of LB broth at 37°C until reaching an OD_600_ value of 0.8. Each well of a sterile 12-well PVC microtiter plate was filled with 2 mL of LB broth and 4 μL of bacterial suspension; 2 mL of LB broth with the 50-fold concentrated root exudate (or 200 μM different composition of root exudates) and 4 μL of bacterial suspension were considered as one treatment. Wells containing only 2 mL of LB broth were used as negative controls. The microtiter plates were incubated at 37°C for 3 days without agitation (Weng et al., 2013).

### Fungal Growth Inhibition Assays

The effect of GUMT319 on hyphal growth was tested using the dual culture method. Interaction experiments were performed using PDA and LB media. An agar plug (0.5 cm diameter) of actively growing fungi was placed at the center of a PDA plate and incubated for 1 day. GUMT319 was then inoculated at two points, each at a distance of 2 cm from the plug, and plates were photographed after 7 days (Li et al., 2014). This experiment was conducted for five replicates.

### Determination of Disease Control Efficacy Under Field Conditions

To determine the disease control efficacy of GUMT319 under field conditions, experiments were conducted during 2018–2020 in a field naturally infested with tobacco black shank disease. The experimental field was located in Meitan, Zunyi (107°41′N, 27°47′E), Guizhou, China. Roots taken from tobacco seedlings of cultivar Yunyan 87 were soaked in GUMT319 suspension (10^8^ CFU/mL) for 1 h and then transplanted. During growth, each tobacco plant was irrigated with 100 mL of GUMT319 suspension (10^8^ CFU/mL) at 7, 14, and 21 days post-transplantation, and the disease index was measured at 50 and 60 days post-transplantation. Due to frequent rain during the growing season, the GUMT319 suspension was applied more often when appropriate. At 21 days post-transplantation, tobacco plants that had been irrigated with 100 mL of 1:400 of 58% metalaxyl manganese zinc WP (Haixun Agri-Biotech Co., Ltd., Shandong, China) and 1:700 of 10^10^ CFU/g *Bacillus* spp. (Green Conway WP, Sino Green Agri-Biotech Co., Ltd., Beijing, China) were used as controls, and plants irrigated with water were designated as blank controls. In 2018 and 2019, the field experiments were performed in an approximately 0.1-ha demonstration area. Tobacco plants were planted in randomized blocks and each block contained 100 plants. Each of the four treatments was replicated four times. In 2020, the field experiments were performed on an approximately 0.67-ha demonstration area and each treatment contained over 2,000 plants. The appearance of tobacco black shank disease symptoms and the cumulative number of infected plants were recorded at 45 days post-transplantation.

The disease incidence was calculated as the percentage of diseased plants relative to the total number of plants growing in each block; evaluation took place when the disease emerged. Disease severity was scored on a scale of 0–9, as follows: 0, no symptoms; 1, less than one-third of the total leaves wilted; 3, one-third to one-half of the total leaves wilted; 5, one-half to two-thirds of the total leaves wilted; 7, more than two-thirds of total leaves wilted; and 9, plant dead. The disease index was calculated using the following equation:

(1) $$Disease\;index=\frac{\left[\left(a\times 0\right)+\left(b\times 1\right)+\left(c\times 3\right)+\left(d\times 5\right)+\left(e\times 7\right)+\left(f\times 9\right)\right]}{\left(a+b+c+d+e+f\right)\times 9}\times 100$$

where *a*, *b*, *c*, *d*, *e*, and *f* are the number of plants in each disease category.

### Data Analysis

Data were statistically analyzed using SPSS v.24.0 (SPSS Inc., Chicago, IL, United States). Mean values of the control and treatment groups were compared using Duncan’s new multiple range test at a significance level of 5% (*P* < 0.05). All percentage data were subjected to arc-sine transformation before statistical analysis.

## Results

### Identification of Strain GUMT319

**FIGURE 1:**
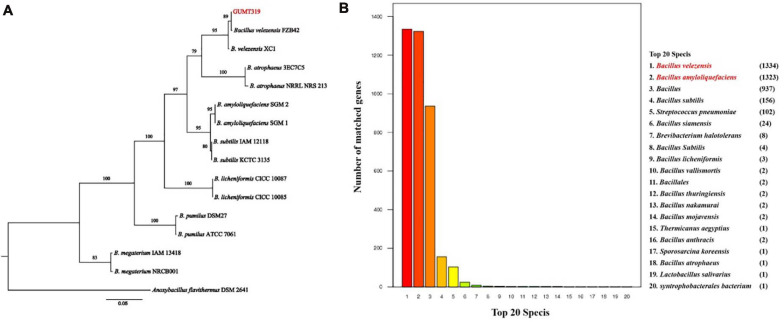
Identification of *Bacillus velezensis* strain GUMT319. **(A)** Phylogenetic tree based on the nucleotide sequences of *16S rRNA* and *gyrA* genes. **(B)** Non-redundant (NR) protein database annotation.

The GUMT319 strain was deposited in the China Center for Type Culture Collection (CCTCC Accession No.: M 2018871). Strain GUMT319 was previously classified as *B. amyloliquefaciens* (Luo et al., 2019). Here, phylogenetic analysis of *16S rRNA* and *gyrA* sequences using the maximum likelihood method placed GUMT319 in a well-supported cluster with *B. velezensis* FZB42 (Figure 1A). BLASTn analysis of *16S rRNA* and *gyrA* sequences amplified from GUMT319 returned a match to the reference strain *B. velezensis* FZB42, with >99% identity. Annotation using the NR protein database indicated that sequences amplified from GUMT319 were most similar to sequences of *B. velezensis* and *B. amyloliquefaciens* (Figure 1B). Thus, taking into account the *16S rRNA* and *gyrA* gene sequences as well as the whole genome sequence comparisons, GUMT319 was identified as a *B. velezensis* strain.

### *B. velezensis* GUMT319 Genome Sequencing and Analysis

**FIGURE 2:**
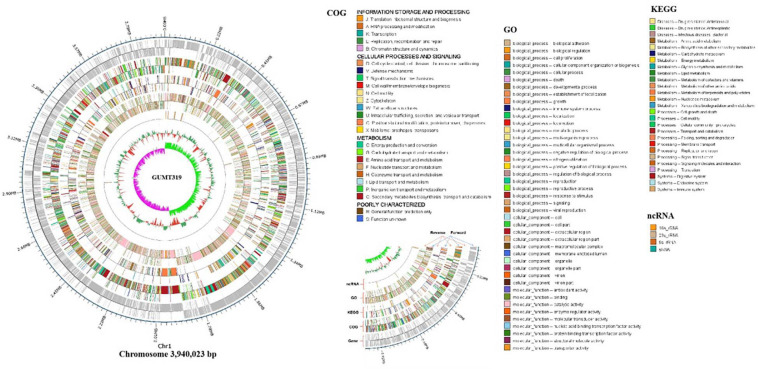
Circular genome map of *B. velezensis* GUMT319. The scale is shown in the outermost circle. The second and third circles indicate genes in forward and reverse orientations, respectively. The outer to inner rings represent forward and reverse DNA sequences of protein-coding genes annotated according to COG, KEGG, and GO databases and ncRNA. The 10th circle shows the GC content, and the 11th circle shows the GC skew in green and purple.

**FIGURE 3:**
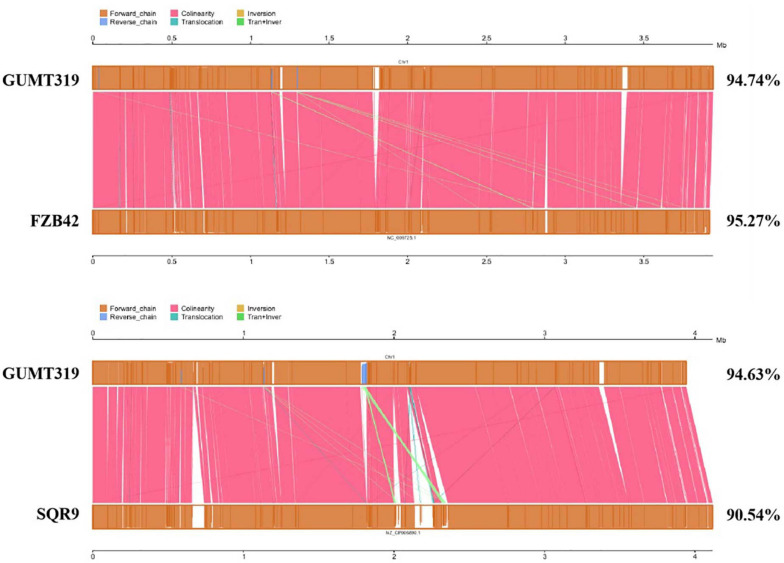
Collinearity analysis of *B. velezensis* GUMT319 and *Bacillus* spp. chromosomes.

To investigate the biocontrol mechanisms of *B. velezensis* GUMT319 and its application for sustainable agriculture, its complete genome sequence was determined. The *B. velezensis* GUMT319 genome consisted of a single circular chromosome 3,940,023 bp in length, with an average GC content of 46.6% (Figure 2), and did not harbor any plasmids. The whole genome of GUMT319 was predicted to contain 4,053 protein-coding genes covering 90.0% of the genome, with an average gene length of 875 bp, as well as 27 rRNAs and 86 tRNAs. Principal features of the genomes of *B. velezensis* GUMT319 and model strains, *B. velezensis* FZB42 and SQR9, are summarized in Table 1 (Chen et al., 2007; Zhang et al., 2015). We performed a collinearity analysis to further compare the genomic similarities and differences between the genome of GUMT319 and those of FZB42 and SQR9 (Figure 3). The results showed that the GUMT319 genome displayed different synteny to those of the other strains. GUMT319 showed the highest synteny with *B. velezensis* FZB42, indicating that their evolutionary stages were the closest, and their genomes were closely related. Amino acid sequence similarity searches against various databases with an E-value threshold of 1e-5 revealed that 3,915 (96.6%), 3,874 (95.6%), 3,293 (81.2%), 2,884 (71.2%), and 2,623 (64.7%) protein-coding genes showed matches in the NR, KEGG, Swiss-Prot, COG, and GO databases, respectively. Abnormal hyphae of plant pathogens were reported in dual cultures with GUMT319, which could be due to the production of secondary metabolites (Luo et al., 2019). Bioinformatics analyses showed that the GUMT319 genome contained 13 putative gene clusters involved in the biosynthesis of secondary metabolites with potential antimicrobial activities (Table 2), most of which were conserved in all *B. velezensis* strains (bacilysin, surfactin, macrolactin, fengycin, bacillaene, difficidin, and terpene; Supplementary Figure 1; Grady et al., 2019). Among these gene clusters, five encoded non-ribosomal peptide synthetases (NRPSs), four encoded trans-acyl transferase polyketide synthetases (transAT-PKSs), and one encoded type III polyketide synthetase (T3PKS), while two clusters were involved in terpene biosynthesis, and one cluster was involved in lantipeptide biosynthesis (Table 2). In contrast, the clusters predicted to produce lantipeptide had not typically been found in other *Bacillus* spp. (Supplementary Figure 1).

**TABLE 1:** Genomic features of *Bacillus velezensis* strains GUMT319, SQR9, and FZB42.

Strains	GUMT319	SQR9	FZB42
Location of isolation	Tobacco rhizosphere	Cucumber rhizosphere	Sugar beet rhizosphere
Genome size (bp)	3,940,023	4,117,023	3,918,596
GC content (%)	46.6	46.1	46.4
No. of protein-coding genes	4,053	3,902	3,687
Percent coding region	89.1	89.0	88.0
No. of rRNA genes	27	21	29
No. of tRNA genes	86	72	88
No. of phage-associated genes	671	218	44
NCBI Accession No.	NZ_CP068563	NZ_CP006890	NC_009725

**TABLE 2:** Antimicrobial gene clusters present in *B. velezensis* GUMT319.

Number	Predicted product	Enzyme complex	Genome location
Cluster 1	Lantipeptide		GUMT319_GM000192-GM000216
Cluster 2	Surfactin	nrps	GUMT319_GM000316-GM000362
Cluster 3	Unknown	Otherks	GUMT319_GM000931-GM000977
Cluster 4	Terpene		GUMT319_GM001059-GM001083
Cluster 5	Macrolactin	Transatpks	GUMT319_GM001461-GM001509
Cluster 6	Bacillaene	Transatpks; nrps	GUMT319_GM001737-GM001789
Cluster 7	Fengycin (iturin)	Transatpks; nrps	GUMT319_GM001911-GM001981
Cluster 8	Terpene		GUMT319_GM002014-GM002037
Cluster 9	Unknown	t3pks	GUMT319_GM002126-GM002183
Cluster 10	Difficidin (polyketide)	Transatpks	GUMT319_GM002319-GM002375
Cluster 11	Bacteriocin	nrps	GUMT319_GM003069-GM003138
Cluster 12	Surfactin (cyclic lipopeptide)	nrps	GUMT319_GM003432-GM003473
Cluster 13	Bacilysin		GUMT319_GM003703-GM003751

### Analysis of GUMT319 Genes Involved in Plant Root Colonization

When *B. velezensis* GUMT319 was grown in liquid culture without agitation, it formed robust pellicles at the liquid–air interface (Luo et al., 2019). The genome of *B. velezensis* GUMT319 contained a complete set of genes implicated in biofilm formation, including 15 genes belonging to the exopolysaccharide (EPS) operon *epsA-O* and *tapA-sipW-tasA* operon, which are required for the production of EPS and TasA fibers, which hold chains of cells together in bundles (Chen et al., 2007; Gao et al., 2015a; Al-Ali et al., 2018; Table 3). *B. velezensis* GUMT319 displayed a robust swarming phenotype (Supplementary Figure 2), and the protein encoded by *swrA* was essential for swarming. The genome of *B. velezensis* GUMT319 contained a complete set of genes implicated in chemotaxis and motility, including six MCPS genes, 25 flagellin gene clusters, and nine chemotaxis protein genes (Liu et al., 2020; Table 3). A previous study shows that GUMT319 produces siderophores, which facilitate rhizosphere competition and growth promotion (Gu et al., 2020). The genome of *B. velezensis* GUMT319 contained genes involved in siderophore biosynthesis, such as *dhbB*, *dhbE*, *dhbF*, and *entC*. GUMT319 also contained master global transcriptional regulators, such as AbrB and Spo0A, as well as the quorum-sensing regulators LuxS/AI-2 and ComA/ComP, which regulate biofilm formation, chemotaxis, and root colonization (Comella and Grossman, 2005; Weng et al., 2013; Yan et al., 2016; Xiong et al., 2020; Table 3).

**TABLE 3:** List of root colonization-associated genes identified in the GUMT319 genome.

Colonization traits	Gene ID	Gene name	Function
Chemotaxis and motility	GUMT319_GM001681	*cheA*	Chemotaxis protein, sensor kinase
	GUMT319_GM001680	*cheB*	Chemotaxis response regulator protein, glutaminase
	GUMT319_GM001683	*cheC*	Chemotaxis protein, CheY-P-specific phosphatase
	GUMT319_GM002228	*cheR*	Chemotaxis protein methyltransferase
	GUMT319_GM001423	*cheV*	Chemotaxis protein
	GUMT319_GM001682	*cheW*	Chemotaxis protein, purine-binding protein
	GUMT319_GM001671	*cheY*	Chemotaxis protein
	GUMT319_GM003009	*tlpB*	Methyl-accepting chemotaxis protein
	GUMT319_GM003010	*mcpA*	Methyl-accepting chemotaxis protein
	GUMT319_GM003011	*tlpA*	Methyl-accepting chemotaxis protein
	GUMT319_GM003012	*mcpB*	Methyl-accepting chemotaxis protein
	GUMT319_GM000730	*yfmS*	Methyl-accepting chemotaxis protein
	GUMT319_GM001414	*mcpC*	Methyl-accepting chemotaxis protein
	GUMT319_GM001382	*motA*	Flagellar motor protein, chemotaxis protein
	GUMT319_GM001381	*motB*	Flagellar motor protein, chemotaxis protein
	GUMT319_GM001655–GM001679	*fla*	Flagellin gene clusters, flagellar biosynthesis
	GUMT319_GM003455	*swrA*	Swarming motility protein
Biofilm	GUMT319_GM000044	*abrB*	Transition state regulatory protein
	GUMT319_GM002412	*spo0A*	Stage 0 sporulation protein A, quorum sensing
	GUMT319_GM002455	*tapA*	Amyloid fiber anchoring and assembly protein, biofilm formation
	GUMT319_GM002454	*sipW*	Signal peptidase, biofilm formation
	GUMT319_GM002453	*tasA*	Spore coat-associated protein, biofilm formation
	GUMT319_GM002452	*sinR*	HTH-type transcriptional regulator, master regulator for biofilm formation
	GUMT319_GM002451	*sinI*	Anti-repressor of *sinR*
	GUMT319_GM003356–GM003370	*epsA-O*	Polysaccharide biosynthesis protein
Siderophores	GUMT319_GM003092	*entC*	Biosynthesis of siderophore group non-ribosomal peptides
	GUMT319_GM003091	*dhbE*	Siderophore group non-ribosomal peptide synthetase
	GUMT319_GM003090	*dhbB*	Biosynthesis of siderophore group non-ribosomal peptides
	GUMT319_GM003089	*dhbF*	Siderophore group non-ribosomal peptide synthetase
Quorum sensing	GUMT319_GM002966	*luxS*	Quorum-sensing autoinducer 2 (AI-2) synthesis protein
	GUMT319_GM003062	*comA*	Two-component transcriptional regulator, quorum sensing
	GUMT319_GM003063	*comP*	Sensor histidine kinase, quorum sensing

### Root Colonization

**FIGURE 4:**
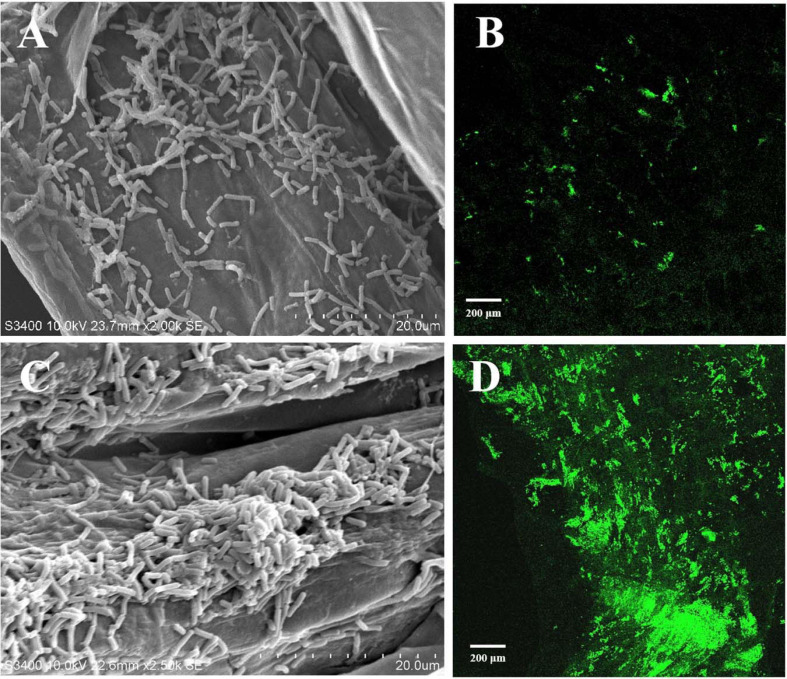
Root colonization assay of *B. velezensis* GUMT319. **(A)** Scanning electron microscope (SEM, HITACHI S-3400N) images of pepper seedling roots colonized by strain GUMT319. **(B)** Confocal laser scanning microscope (CLSM, Olympus FV10i) images of pepper seedling roots colonized by GUMT319-gfp. **(C)** SEM images of tobacco seedling roots colonized by strain GUMT319. **(D)** Confocal laser scanning microscope (CLSM) images of tobacco seedling roots colonized by GUMT319-gfp. The green fluorescence signal depicts live cells colonizing the tobacco roots.

Root colonization of the rhizosphere by antagonistic bacteria is a prerequisite for effective biological control (Liu et al., 2014). In this study, we investigated the colonization of healthy tobacco and pepper seedling roots by *B. velezensis* GUMT319. After 2 days of incubation in the hydroponic system, root colonization by *B. velezensis* GUMT319 and GUMT319-gfp was investigated using SEM and a CLSM. The SEM images showed that GUMT319 cells were rod-shaped (Figures 4A,C). Additionally, a complex biofilm structure consisting of GUMT319 cells (Figure 4C) and GUMT319-gfp cells (Figure 4D) was formed on the tobacco root surface, and colonization occurred preferentially in the elongation region of tobacco roots (Figure 4). We also found that GUMT319 (Figure 4A) and GUMT319-gfp (Figure 4B) could colonize pepper roots, although to a much lesser extent than in tobacco roots (Figure 4).

### Chemotactic Response to Root Exudates and Biofilm Formation

Root exudates are known to play an important role in plant–microbe interaction in the rhizosphere. The different compositions of root exudates from different plants are directly related to the chemotaxis reaction, biofilm formation, and colonizing behavior of bacterial strains originating from the respective rhizosphere (Zhang et al., 2014). We wanted to identify the different components of the root exudates that mediate the interaction between biocontrol *Bacillus* spp. and plant roots, which influenced their root colonization. To this end, root exudates from tobacco and pepper plants were collected and analyzed. Qualitative analyses of the freeze-dried tobacco and pepper root exudates were performed by GC-TOF-MS, and 248 peaks (Supplementary Figure 2) were detected, including amino acids, organic acids, and others (data not shown). Here, some organic acids (cinnamic acid, fumaric acid, phthalic acid, benzoic acid, and lauric acid) and nicotine were selected as the targets for evaluation of their roles on *B. velezensis* GUMT319 (Zhang et al., 2014; Feng et al., 2018; Ma et al., 2018). Cinnamic acid, fumaric acid, and benzoic acid were detected both in tobacco and pepper root exudates. Phthalic acid and nicotine were found only in tobacco root exudates and lauric acid was found only in pepper root exudates.

**FIGURE 5:**
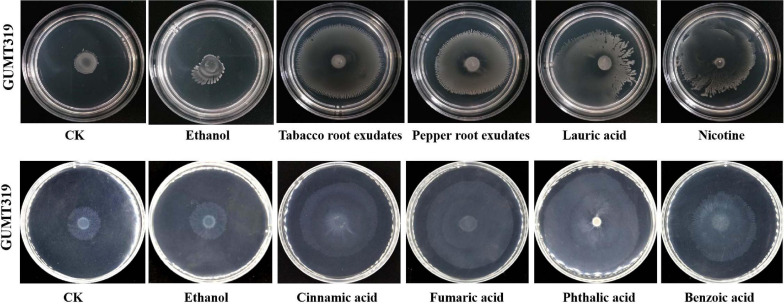
Chemotactic response of *B. velezensis* GUMT319 to root exudates.

**FIGURE 6:**
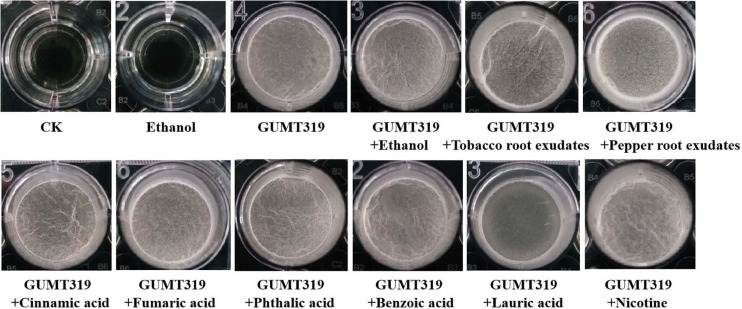
Biofilm formation assay of *B. velezensis* GUMT319.

To investigate the cause of root colonization by *B. velezensis* GUMT319, the chemotactic reaction and biofilm formation response of GUMT319 to two root exudates and six components were determined. After the 12-h incubation period, the LB (0.7% agar) plate was almost fully covered by GUMT319 cells (Supplementary Figure 2), indicating that GUMT319 was highly proficient in swarming. We measured the chemotaxis activity of strain GUMT319 using tobacco root exudates, pepper root exudates, organic acids (cinnamic acid, fumaric acid, phthalic acid, benzoic acid, and lauric acid), and nicotine as attractants. The root exudates induced a positive chemotactic response in *B. velezensis* GUMT319, and cells showed faster migration toward all the attractants than toward the control after a 6-h incubation (Figure 5). The biofilm formation activity of strain GUMT319 was also examined using the qualitative biofilm experiment with tobacco root exudates, pepper root exudates, organic acids (cinnamic acid, fumaric acid, phthalic acid, benzoic acid, and lauric acid), and nicotine as attractants. The results showed that only lauric acid could be involved in the negative regulation of biofilm formation. No obvious difference was observed in biofilm formation among other treatments conducted with or without root exudates. The pellicles formed by GUMT319 were similar in all other groups (Figure 6).

### Antagonistic Effects of GUMT319 on Plant Pathogens *in vitro*

**FIGURE 7:**
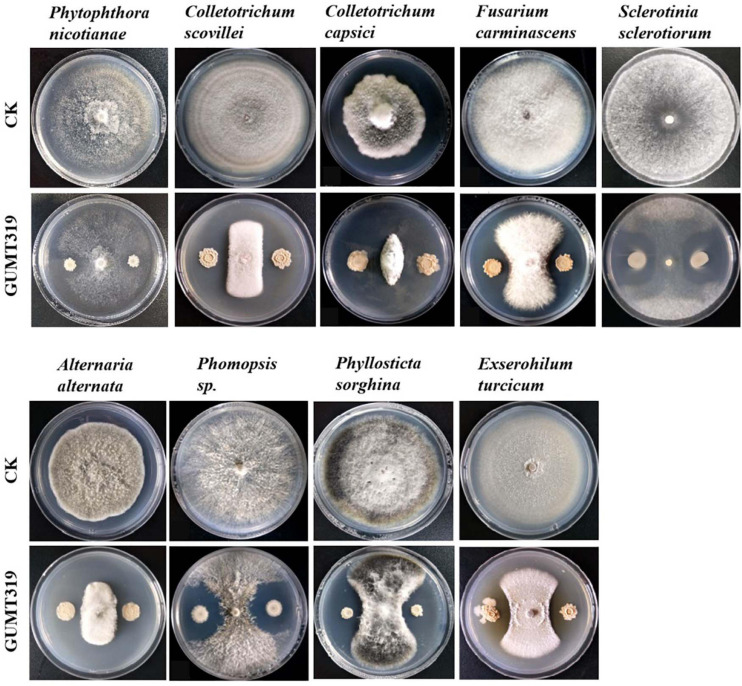
Antagonistic activity of *B. velezensis* GUMT319 against nine plant pathogens in dual-culture test.

In this study, it was found that GUMT319 displayed strong antagonistic activity against *P. nicotianae*, *C. scovillei*, *C. capsici*, *F. carminascens*, *S. sclerotiorum*, *A. alternata*, *Phomopsis* sp., *P. sorghina*, and *E. turcicum* (Figure 7 and Supplementary Table 2), indicating that *B. velezensis* GUMT319 was effective against a relatively broad spectrum of oomycetes and ascomycetes.

### Effects of GUMT319 on Tobacco Black Shank in Field Experiments

We further investigated the efficiency of control of GUMT319 on tobacco black shank in field experiments. In those conducted in 2018, the disease incidence of tobacco plants treated with sterile water was 20.48%, while the disease incidence of plants treated independently with GUMT319 suspension, 58% metalaxyl manganese zinc WP, and Green Conway WP decreased, having control efficacy on tobacco black shank of 77.15, 76.43, and 65.59%, respectively (Table 3). Tobacco plants treated with strain GUMT319 showed the lowest disease incidence, with a control efficiency significantly higher than those treated with Green Conway WP. In the 2019 field experiments, the disease incidence of tobacco plants treated with water was 15.42%, while the control efficacy on tobacco black shank of those plants treated independently with GUMT319, 58% metalaxyl manganese zinc WP, and Green Conway WP were 71.97, 71.59, and 66.47%, respectively (Table 3). In the 2020 field experiments, the disease incidence of tobacco plants treated with water reached 79.09%, while the control efficacy on tobacco black shank of those plants treated independently with GUMT319, 58% metalaxyl manganese zinc WP, and Green Conway WP were 79.54, 76.98, and 62.31%, respectively (Table 4 and Supplementary Figure 4). Thus, the control effect of GUMT319 suspension was significantly higher than that of 58% metalaxyl manganese zinc WP and Green Conway WP (*P* < 0.05), suggesting that GUMT319 could be used as a potential BCA for tobacco black shank.

**TABLE 4:** Control efficacy of strain GUMT319 against tobacco black shank in the field.

Treatments	2018			2019			2020		
	Disease incidence (%)	Disease index	Control efficacy (%)	Disease incidence (%)	Disease index	Control efficacy (%)	Disease incidence (%)	Disease index	Control efficacy (%)
Water (control)	20.48 ± 1.52a	5.93 ± 0.24a	–	15.42 ± 0.44a	5.14 ± 0.09a	–	79.09 ± 0.48a	19.57 ± 0.13a	–
GUMT319	5.26 ± 0.63b	6.17 ± 0.18c	77.15 ± 2.60a(A)	5.17 ± 0.81b	1.48 ± 0.15b	70.97 ± 2.98a(A)	11.66 ± 0.16d	4.01 ± 0.04d	79.54 ± 0.18a(A)
58% metalaxyl manganese zinc WP	7.16 ± 0.92b	1.20 ± 0.13c	76.43 ± 3.48a(A)	4.63 ± 0.39b	1.45 ± 0.08b	71.59 ± 1.60a(A)	22.61 ± 0.43c	4.51 ± 0.11c	76.98 ± 0.54b(B)
Green Conway WP	8.65 ± 2.86b	1.76 ± 0.14b	65.59 ± 2.78b(A)	4.95 ± 0.22b	1.71 ± 0.07b	66.47 ± 1.37a(A)	43.68 ± 0.37b	7.39 ± 0.08b	62.31 ± 0.43c(C)

*The data are means ± SDs. The different lowercase and uppercase letters in the same column indicate significant difference at the P < 0.05 and P < 0.1 levels, respectively, using Duncan’s new multiple range test. All percentage data were subjected to arc-sine transformation before statistical analysis.*

## Discussion

The tobacco black shank control efficiency of several *Bacillus* strains, such as *B. subtilis* Tpb55 and *B. velezensis* Ba168, has been tested previously. Tobacco black shank, caused by *P. nicotianae*, is destructive to almost all flue-cured tobacco cultivars and is widespread in many tobacco-growing countries. *Bacillus* strains represent a promising strategy for the management of this soil-borne disease (Han et al., 2016; Zhang et al., 2017a; Srikhong et al., 2018; Guo et al., 2020). In this study, we identified another *B. velezensis* isolate, GUMT319, from a healthy tobacco rhizosphere in the fields in Guizhou with a high incidence of tobacco black shank. The control efficiency of GUMT319 against tobacco black shank was greater than 70% in field experiments conducted in Meitan in 2018–2020, which was significantly higher than that obtained using other treatments including fungicide application (Table 4 and Supplementary Figure 4). Hence, GUMT319 could potentially be used as a BCA for tobacco black shank in Guizhou.

GUMT319 was previously identified as a *B. amyloliquefaciens* strain; however, *16S rRNA* and *gyrA* sequences and whole genome sequence comparisons in the current study revealed that GUMT319 is a *B. velezensis* strain (Figure 1). Similarly, SQR9 and FZB42, which were previously classified as *B. amyloliquefaciens* strains, are now recognized as *B. velezensis* strains and considered as the model strains for gram-positive plant growth-promoting and biocontrol rhizobacteria (Chen et al., 2007; Zhang et al., 2015; Fan et al., 2018). We support the theory that many strains currently classified as *B. amyloliquefaciens* are actually *B. velezensis*.

In this study, we identified and classified *B. velezensis* strain GUMT319 based on genomic data generated using second- and third-generation sequencing technologies. Strain GUMT319 contained a single circular chromosome 3,940,023 bp in length, with 4,053 predicted genes and an average GC content of 46.6% (Figure 2). The genome of GUMT319 is smaller than that of SQR9 (4,117,023 bp) but comparable to that of FZB42 (3,918,596 bp). The three strains were identical in terms of the number of protein-coding genes but differed in the number and size of prophage regions as well as gene clusters encoding secondary metabolites. The majority of genes unique to GUMT319 (i.e., absent in SQR9 and FZB42) were phage related (Table 1 and Figure 3).

The ability of *B. velezensis* GUMT319 to efficiently colonize the surface of tobacco roots was a prerequisite for biocontrol. Many beneficial bacteria form biofilms on plant roots, which play an important role in their antagonistic activities and biocontrol efficacy (Chen et al., 2013). Rhizosphere competence of bacteria is associated with their ability to form sessile, multicellular communities (biofilms) and their chemotaxis motility (Niu et al., 2017; Liu et al., 2020). The genome of GUMT319 contains more than 60 genes and 13 putative gene clusters related to secondary metabolites, which have previously been described as being involved in biofilm formation, chemotaxis motility, growth promotion, and antifungal activity (Tables 2, 3).

*B. velezensis* has been widely researched because of its potential to produce bacteriostatic secondary metabolites (Fan et al., 2018). *B. velezensis* strain GUMT319 has been reported to produce cell wall-degrading enzymes such as proteases, cellulases, and phosphatases as well as siderophores, which facilitate rhizosphere competition and plant growth promotion and show strong inhibitory activity against *P. nicotianae* as well as eight other plant pathogens *in vitro* (Luo et al., 2019). The GUMT319 genome contained 13 putative gene clusters that were expected to participate in antimicrobial production, including bacilysin, surfactin, macrolactin, fengycin, bacillaene, difficidin, and terpene (Table 3 and Supplementary Figure 1; Grady et al., 2019); the majority of these gene clusters are conserved in all *B. velezensis* strains. However, clusters predicted to produce lantipeptides were not typical in other *Bacillus* spp. (Supplementary Figure 3). Lantipeptides are ribosomally synthesized peptides that are extensively post-translationally modified. It is suggested that lantipeptides are the antimicrobial compounds produced by gram-positive bacteria (Grady et al., 2019).

The ability of *Bacillus* to colonize plant roots and proliferate in the rhizosphere is important for stable, long-lasting disease prevention (Han et al., 2016). To examine the ability of GUMT319 to colonize plant roots, we labeled the strain with GFP. In the present study, GUMT319 and GUMT319-gfp colonized the surface of tobacco roots, forming microcolonies and complex biofilm structures. The root elongation zone was colonized to a greater extent than other areas of the root. We also found that GUMT319 and GUMT319-gfp could colonize on pepper roots, but lesser than that on tobacco roots (Figure 4). As a signal to attract or repel microbes, the root exudates serve as a carbon source for soil microorganisms. In addition, they play significant roles in chemotaxis, biofilm formation, and colonization of *Bacillus* strains on plant roots (Feng et al., 2018). The composition of root exudates is closely related to plant species, soil environment, climate factors, microorganisms, nutritional status, etc. Among them, plant species is the most important (Zhang et al., 2014). Therefore, we speculated that some substances in tobacco root exudates (not pepper) play a key role. The root exudates from tobacco and pepper plants were collected and analyzed using GC-TOF-MS (Supplementary Figure 3). It was found that organic acids were the most represented class of compounds in both plants. Hence, some organic acids (cinnamic acid, fumaric acid, phthalic acid, benzoic acid, and lauric acid) (Zhang et al., 2014; Feng et al., 2018) and nicotine (Ma et al., 2018) were selected as targets for the evaluation of their roles on *B. velezensis* GUMT319. Cinnamic acid, fumaric acid, and benzoic acid were detected both in tobacco and pepper root exudates, phthalic acid and nicotine in tobacco root exudates, and lauric acid only in pepper root exudates.

In this study, in tobacco root exudates, pepper root exudates, organic acids (cinnamic acid, fumaric acid, phthalic acid, benzoic acid, and lauric acid), and nicotine as attractants, it was found that GUMT319 moved faster toward all root exudates than toward the control (Figure 5). Lauric acid could be involved in the negative regulation of biofilm formation. There was no obvious difference among other treatments conducted with or without root exudates in biofilm formation (Figure 6). Compared with other root-secreted compounds, it was first reported that nicotine in the root exudates of tobacco was a chemoattractant for *B. velezensis* GUMT319. Previous research shows that nicotine from tobacco root exudates has the ability to enhance chemotaxis, growth, biocontrol efficiency, and colonization by *Pseudomonas aeruginosa* NXHG29 (Ma et al., 2018). However, we do not know how *B. velezensis* GUMT319 recognizes nicotine and regulates chemotaxis and root colonization.

Overall, this study showed that the beneficial function of GUMT319 in controlling tobacco black shank disease is through its ability to inhibit the mycelial growth of plant pathogens and to successfully colonize tobacco roots. Hence, *B. velezensis* GUMT319 could be used as a potential BCA against tobacco black shank.

## Data Availability Statement

The datasets presented in this study can be found in online repositories. The names of the repository/repositories and accession number(s) can be found below: https://www.ncbi.nlm.nih.gov/genbank/, CP068563.

## Author Contributions

HD, LP, and ZL designed the research and wrote the manuscript. HD, WM, SY, HC, and LP performed all the experiments. HD and WM analyzed the data. All authors reviewed the final manuscript.

## Conflict of Interest

The authors declare that the research was conducted in the absence of any commercial or financial relationships that could be construed as a potential conflict of interest.
