# Supplementary material for: Whole Genome Sequence of Bacillus velezensis Strain GUMT319: A Potential Biocontrol Agent Against Tobacco Black Shank Disease
Source: Front Microbiol. 2021 Jul 6;12:658113. doi: 10.3389/fmicb.2021.658113 (PMC8291047; doi:10.3389/fmicb.2021.658113)
Supplement: Supplementary file 1 [file Data_Sheet_1.docx]

Supplementary Material

# Supplementary Figures and Tables

## Supplementary Figures


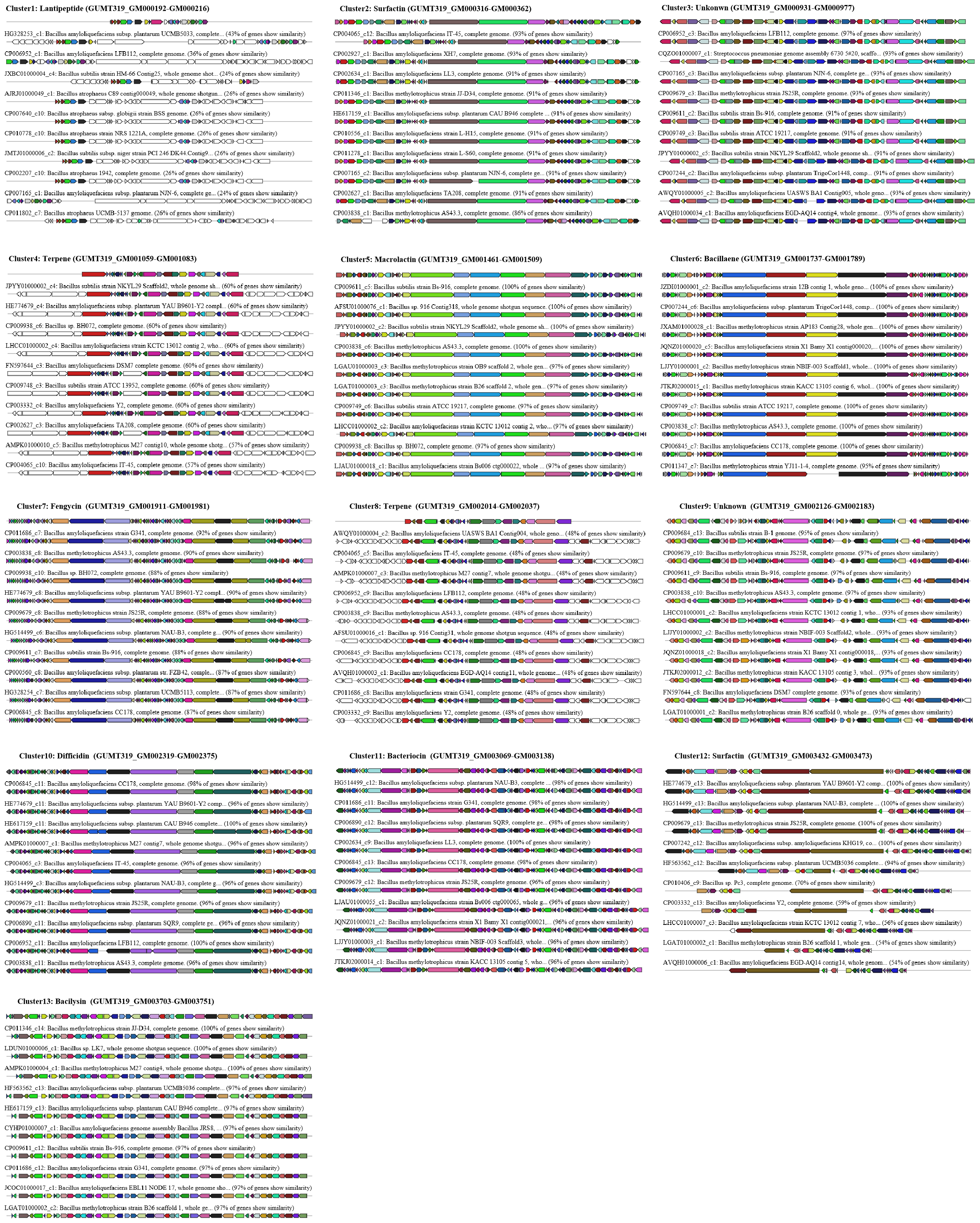


**Supplementary Figure 1.** Gene cluster blast comparative analysis.


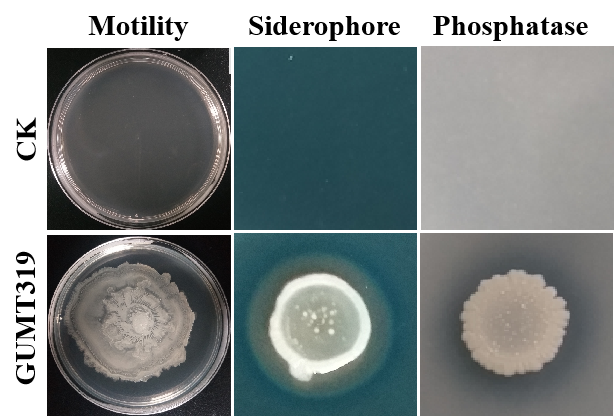


**Supplementary Figure 2.** Detection of motility ,siderophore and phosphatase activity in *Bacillus velezensis* GUMT319.


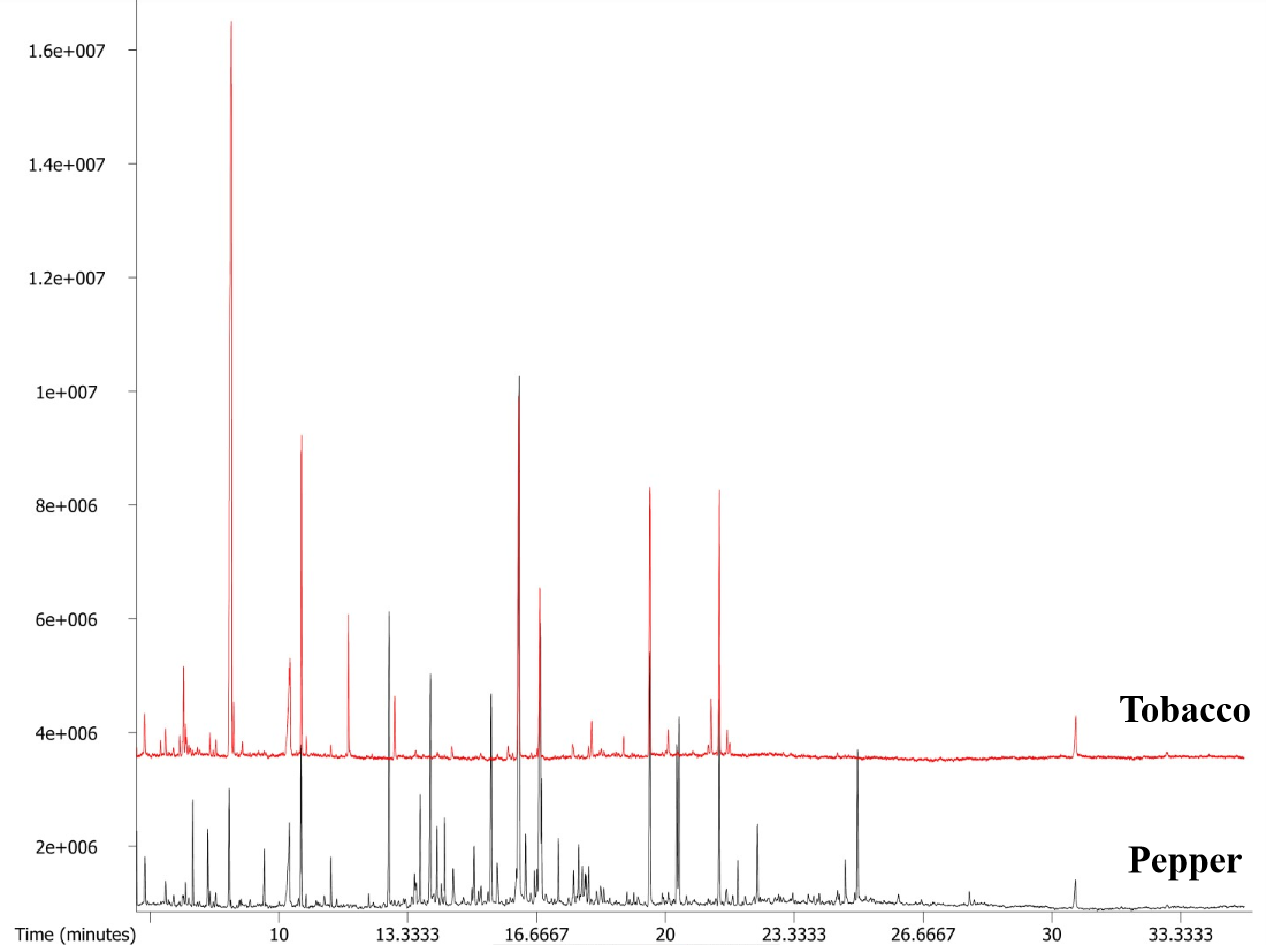


**Supplementary Figure 3.** Qualitative analyses of freeze-dried tobacco and pepper root exudates by GC-TOF-MS.


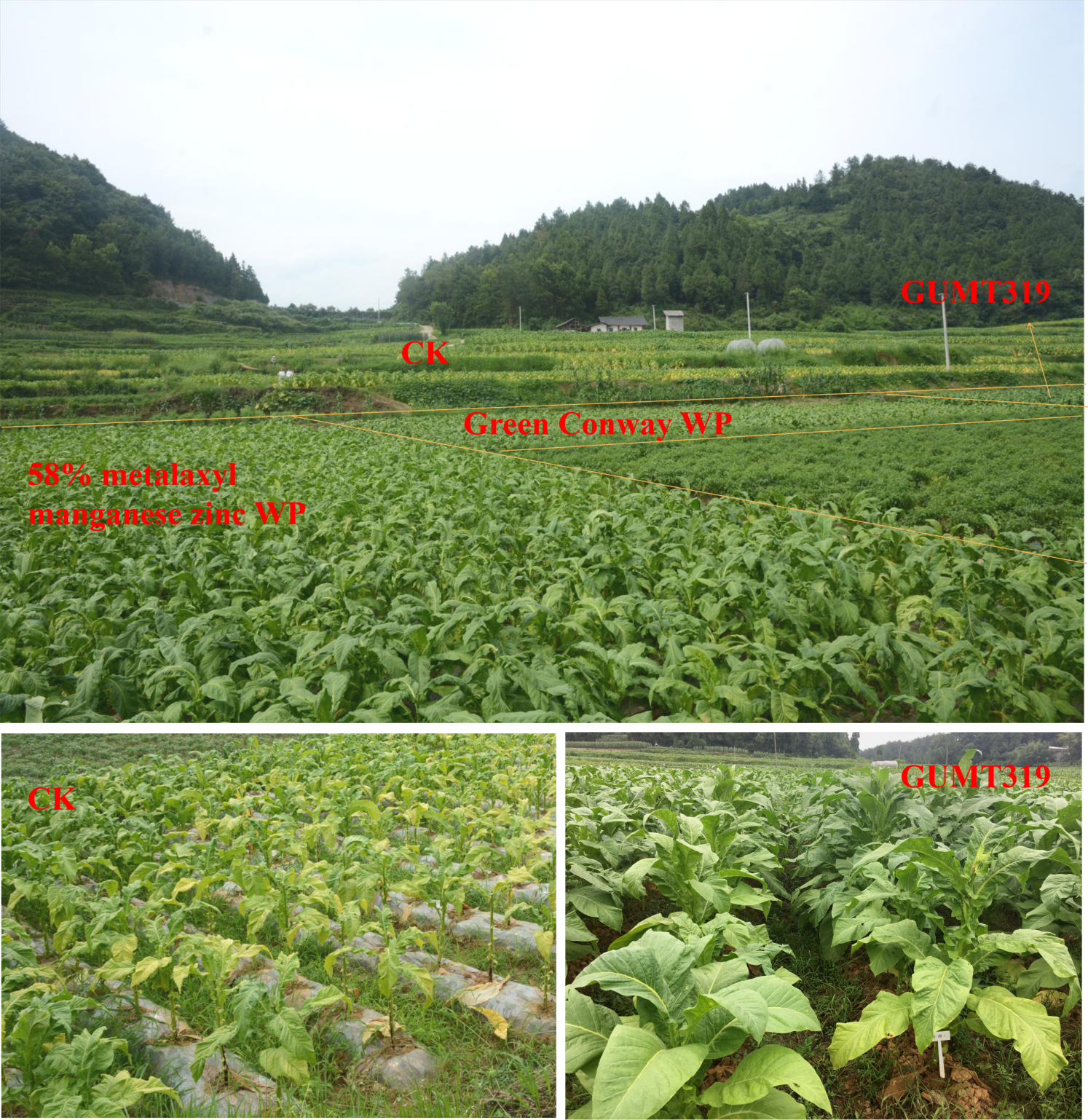


**Supplementary Figure 4.** Control efficacy of strain GUMT319 against tobacco black shank in the field in 2020.

## Supplementary Tables

**Supplementary Table 1.** *Bacillus* species used for phylogenetic analysis, along with their corresponding GenBank accession numbers.

| Species | Accession No. | GenBank Accession No. | |
| --- | --- | --- | --- |
|  |  | *16S rRNA* | *gyrA* |
| *Anoxybacillus flavithermus* | DSM 2641* | NR114414 |  |
| *Bacillus amyloliquefaciens* | SGM-1 | MT427748 | MW070537 |
| *B. amyloliquefaciens* | SGM-2 | MT427749 | MW070538 |
| *Bacillus atrophaeus* | 3EC7C5 | EU304976 | EF026698 |
| *B. atrophaeus* | NRRL NRS 213* | NR116190 | EF026731 |
| *Bacillus licheniformis* | CICC 10085 | GQ375230 | GQ355995 |
| *B. licheniformis* | CICC 10087 | GQ375232 | GQ355997 |
| *Bacillus pumilus* | ATCC 7061* | NR043242 |  |
| *B. pumilus* | DSM27 | AM237370 | JX513936 |
| *Bacillus megaterium* | IAM 13418* | NR043401 |  |
| *B. megaterium* | NRCB001 | MN128363 | MN662261 |
| *Bacillus sonorensis* | NRRL B 23157 | AF302121 | EF026730 |
| *B. sonorensis* | R 19056 | AJ586363 | AJ586404 |
| *Bacillus subtilis* | IAM 12118* | MK267098 |  |
| *B. subtilis* | KCTC 3135 | FJ917386 | EF538682 |
| *Bacillus velezensis* | FZB42 | KX898131 | ABS72455 |
| *B. velezensis* | XC1 | MT649755 | MT683381 |

**Supplementary Table 2.** Antagonistic activity of *B. velezensis* GUMT319 against nine plant pathogens in dual-culture test.

| Pathogens | Diameter of fungal growth (mm ± SD) | | Inhibition rate (%) |
| --- | --- | --- | --- |
|  | CK | GUMT319 |  |
| *Phytophthora nicotianae* | 76.3±5.8 | 31.5±1.6 | 58.7 |
| *Colletotrichum scovillei* | 80.3±2.6 | 23.6±1.0 | 70.6 |
| *Colletotrichum capsici* | 56.7±3.1 | 14.6±3.4 | 74.3 |
| *Fusarium carminascens* | 74.1±0.1 | 27.7±1.2 | 62.6 |
| *Sclerotinia sclerotiorum* | 82.6±0.3 | 25.5±1.5 | 69.1 |
| *Alternaria alternata* | 55.2±2.1 | 24.1±1.3 | 56.3 |
| *Phomopsis*sp*.* | 80.9±1.2 | 19.3±2.2 | 76.1 |
| *Phyllosticta sorghina* | 72.0±4.2 | 24.4±1.4 | 66.1 |
| *Exserohilum turcicum* | 81.2±2.1 | 23.5±0.7 | 71.1 |
